# Switching industrial production processes from complex to defined media: method development and case study using the example of *Penicillium chrysogenum*

**DOI:** 10.1186/1475-2859-11-88

**Published:** 2012-06-22

**Authors:** Andreas E Posch, Oliver Spadiut, Christoph Herwig

**Affiliations:** 1 Institute of Chemical Engineering, Research Area Biochemical Engineering, Gumpendorfer Straße 1a, Vienna University of Technology, A-1060, Vienna, Austria

**Keywords:** Filamentous fungi, Complex media, Defined media, Stoichiometric mass balancing, Fast strain characterization

## Abstract

**Background:**

Filamentous fungi are versatile cell factories and widely used for the production of antibiotics, organic acids, enzymes and other industrially relevant compounds at large scale. As a fact, industrial production processes employing filamentous fungi are commonly based on complex raw materials. However, considerable lot-to-lot variability of complex media ingredients not only demands for exhaustive incoming components inspection and quality control, but unavoidably affects process stability and performance. Thus, switching bioprocesses from complex to defined media is highly desirable.

**Results:**

This study presents a strategy for strain characterization of filamentous fungi on partly complex media using redundant mass balancing techniques. Applying the suggested method, interdependencies between specific biomass and side-product formation rates, production of fructooligosaccharides, specific complex media component uptake rates and fungal strains were revealed. A 2-fold increase of the overall penicillin space time yield and a 3-fold increase in the maximum specific penicillin formation rate were reached in defined media compared to complex media.

**Conclusions:**

The newly developed methodology enabled fast characterization of two different industrial *Penicillium chrysogenum* candidate strains on complex media based on specific complex media component uptake kinetics and identification of the most promising strain for switching the process from complex to defined conditions. Characterization at different complex/defined media ratios using only a limited number of analytical methods allowed maximizing the overall industrial objectives of increasing both, method throughput and the generation of scientific process understanding.

## Background

Filamentous fungi have been used for the large scale production of antibiotics, organic acids, enzymes and other industrially relevant compounds for many decades [1,2]. However, due to the increased complexity of filamentous fungal cultivation systems compared to unicellular yeast or bacterial systems, including enhanced media demands during spore germination, adherent wall growth, extracellular proteolytic activity, formation of various side-products and complex growth morphology, process engineers still commonly rely on extensive experience and empiricism during bioprocess development and control rather than using science-based approaches [3,4].

Lacking scientific understanding about interdependencies of critical process parameters (CPP), critical quality attributes (CQA) and key performance parameters (KPP), manufacturers conventionally stick to well-established complex media fermentation protocols to ensure growth and productivity, and thus fail at demonstrating scientific understanding for their thoroughly, though mostly empirically optimized, high-yielding production processes. However, considerable lot-to-lot variability of complex media ingredients not only demands for exhaustive incoming components inspection and quality control, but unavoidably affects process stability and performance [5]. Additionally, the use of complex media components prevents stoichiometric and physiological determination of the bioprocess, resulting in uncontrolled process conditions that inhibit timely accurate detection of physiological changes, as well as the application of appropriate control strategies. Unarguably, hardly controllable process conditions (whether or not due to complex raw materials) inevitably reduce process yields compared to optimally controlled conditions. Moreover, not only the variability of complex raw materials, but also regulatory guidelines in context with the Quality by Design (QbD) initiative issued by the U.S. Food and Drug Administration strongly encourage biopharmaceutical manufacturers to move away from traditional empirically based bioprocess development towards science-based process design and control [6]. As a direct consequence, bioprocesses using complex media ingredients should be switched to defined media.

Up to date, proposed methods for switching microbial production processes to defined media include statistical experimental designs aiming at media optimization [7,8] as well as detailed analysis of complex media components and substitution of the relevant key components in a chemically defined form [9]. Another interesting approach suggests chemical defined media formulation based on flux analysis through the metabolic network reconstructed from the organisms genome sequence [10]. However, these approaches were only described for simple unicellular organisms and not yet for the much more complex fungi which exhibit more complicated strain specific nutrient demands for growth, productivity and germination.

Unfortunately, the most straight-forward solution for switching fungal strains from complex to defined media, namely a simple parallelized strain characterization on defined media, would most likely not only result in a lack of growth for most of the screened fungal strains and thus only yield limited scientific process understanding, but also in highly increased time demands compared to characterization procedures on complex media. Besides, characterization on fully complex media can only be accomplished using exhaustive, time-consuming offline analysis procedures including acid hydrolysis of insoluble nitrogen species as well as macromolecular soluble species [11]. Moreover, having inoculated the fermentation process, insoluble media components cannot be separated from biomass elements and are therefore not quantifiable. Encouraged by these inherent problems in bioprocess analysis for complex, insoluble media compounds, we developed a simple strategy to infer strain specific complex media components uptake kinetics by applying a combined complex/defined media fermentation strategy.

The approach of investigating macroscopic balances for stoichiometric bioprocess modeling was presented already over 30 years ago [12,13]. Over the last decade, we could successfully demonstrate general method applicability for batch, fed-batch and continuous cultivations of bacterial and yeast systems [14-16]. So far however, method dissemination to filamentous fungi and complex media fermentation has been hampered by the difficulty to accurately determine the biomass dry weight concentration for adherently growing organisms in cultivations containing insoluble media components, and the increased complexity of filamentous fungal systems.

In this work, we present a methodology for fast strain characterization of filamentous fungi in batch cultivations on combined complex/defined media. This approach allows the identification of the most promising candidate strain(s) for switching the bioprocess from complex to fully defined media. In the context of this study, method applicability was demonstrated by characterizing two different industrial *Penicillium chrysogenum* candidate strains. The developed methodology is based on minimal analytical needs and the indirect determination of specific uptake rates of complex media components by applying statistically verified redundant mass balancing techniques for carbon, nitrogen and electron balances. Moreover, we propose a strategy facilitating accurate determination of biomass dry weight concentrations throughout the process by successfully preventing adherent wall growth via headspace cooling and keeping the liquid level constant by refilling the withdrawn volume during sampling.

Using the suggested approach for strain characterization in batch cultivations on partly complex media and elucidation of strain specific physiological parameters for complex media components, we could demonstrate accurate and feasible bioprocess characterization for industrial applications at reduced analytical demands in accordance with QbD principles.

## Results and discussion

### Theory and modeling

**Figure 1:**
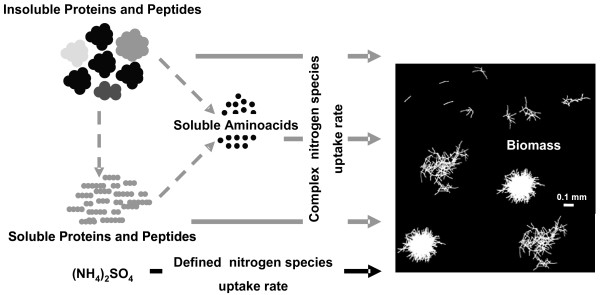
**Nitrogen metabolism for cultivations containing complex and defined nitrogen sources.** Uptake rates for defined nitrogen components can be determined by simple enzymatic methods, uptake rates for complex nitrogen constituents can be inferred from stoichiometric balancing using carbon, degree of reduction and nitrogen balances.

Corn steep liquor and ammonium sulfate were supplied as nitrogen sources in the present study and fast strain characterization was performed based on measured and balanced rates for complex and defined nitrogen uptake (Figure 1). While detailed analysis of complex nitrogen uptake mechanisms includes determining uptake rates of proteins, peptides and amino acids, the here presented approach is based on a simplified model for nitrogen uptake, only accounting for the total combined uptake of complex and defined nitrogen components. In this study we introduce a novel strain specific key parameter, namely the percentage of complex nitrogen of the overall nitrogen uptake (POCN; Eq. 1), which was used for fast and feasible strain characterization on partly complex media.

(1) $$Percentage\;Of\;Complex\;Nitrogen(POCN)=\frac{r_{complexnitrogensources}(Nmol/gBDW/h)}{r_{allnitrogensources}(Nmol/gBDW/h)}$$

Batch cultures in this study can be described by a stoichiometric process equation (Eq.2). Sugars, oxygen as well as defined and complex nitrogen sources are consumed, while biomass, carbon dioxide and side-products are produced.

(2) $$\begin{array}{l} \sum_{I=1}^{n}CH_{a,I}O_{b,I}+Y_{O2/S}O\_2+Y_{NH4/S}NH_{4}^{+}+Y_{ComplexN/S}ComplexN\to \\ Y_{X/S}CH_{\mathrm{xH}}O_{\mathrm{xO}}N_{\mathrm{xN}}+Y_{CO2/S}CO_{2}+\sum_{I=1}^{n}Y_{sideproduct,I/S}CH_{c,I}O_{d,I} \end{array}$$

As concentrations, uptake rates and yields for defined media components can be determined by standard analysis methods, uptake rates and yields for non-measurable media components may be inferred from mass balancing according to the following equations. Calculations of carbon, degree of reduction and nitrogen balances as well as recovery coefficients are described in equations 3 to 11.

### Carbon balance

(3) $$N_{C,IN}=\sum_{I=1}^{n}\Delta N_{substrate,I,IN}$$

(4) $$N_{C,OUT}=\Delta N_{\mathrm{biomass}}+\Delta N_{CO2}+\sum_{I=1}^{n}\Delta N_{metabolite,I,OUT}$$

(5) $$R_{c}=\frac{N_{C,OUT}}{N_{C,IN}}$$

### Degree of reduction balance

(6) $$DOR_{\mathrm{IN}}=\sum_{I=1}^{n}\Delta N_{substrate,I,IN}*dor_{substrate,I}+\Delta N_{O2}*dor_{O2}$$

(7) $$DOR_{\mathrm{OUT}}=\sum_{I=1}^{n}\Delta N_{metabolite,I,OUT}*dor_{metabolite,I}+\Delta N_{\mathrm{biomass}}*dor_{\mathrm{biomass}}$$

(8) $$R_{\mathrm{DOR}}=\frac{DOR_{\mathrm{OUT}}}{DOR_{\mathrm{IN}}}$$

### Nitrogen balance

(9) $$N_{N,IN}=\sum_{I=1}^{n}\Delta N_{substrate,I,IN}*x_{n,I}$$

(10) $$N_{N,OUT}=\Delta N_{\mathrm{biomass}}*x_{n,biomass}$$

(11) $$R_{N}=\frac{N_{N,OUT}}{N_{N,IN}}$$

For all experiments, incremental consumption rates for oxygen, carbohydrates, amino acids, ammonium sulphate as well as evolution rates for carbon dioxide, biomass and side-metabolites, such as gluconic acid or mannitol, were calculated from raw data. As the only unmeasured rate was the uptake rate of complex media constituents, and thus amino acids, the number of the overall determined rates (N) exceeded the number of measured rates (M) by one for fermentations including corn steep liquor while M equalled N for cultivations in defined media. Imposing three balances (K = 3) the degree of redundancy (S) was 3 for defined cultivations and 2 for complex cultivations (S = M + K – N). Calculation of unmeasured rates and data reconciliation to the most probable values was performed in order to fulfill the constraint of mass conservation throughout the process illustrated in equation 12.

(12) E*x=0

where

(13) $$x=\left(\begin{array}{c} rtotalSugars\\ rO_{2}\\ rNH_{4}^{+}\\ rComplexN\\ rX\\ rCO_{2}\\ rMannitol\\ rGluconicAcid\\ rOligosaccharides \end{array}\right)$$

(14) $$E=\left(\begin{array}{cccccccccc} & totalSugars & O_{2} & NH_{4}^{+} & ComplexN & X & CO_{2} & Mannitol & GluconicAcid & Oligossaccharides\\ C & 1 & 0 & 0 & 3.69 & 1 & 1 & 1 & 1 & 1\\ DOR & 4 & -4 & 3 & 18.59 & xDOR & 0 & 4.33 & 3.66 & 4\\ N & 0 & 0 & 1 & 1 & xN & 0 & 0 & 0 & 0 \end{array}\right)$$

For equation 12, *E* is a three times N matrix summarizing parameters for carbon, degree of reduction and nitrogen balances and *x* an N times one vector containing the rates (Eq. 13 and 14). In Eq. 13 units in the rate vector *x* for *totalSugars*, *X*, *CO*_*2*_, *Mannitol*, *GluconicAcid* and *Oligossaccharides* are Cmol/l/h, Nmol/l/h for the nitrogen sources *NH*_*4*_^*+*^ and *ComplexN* and mol/l/h for *O*_*2*_, respectively. For determination of complex media constituents uptake rates, we assumed equal molar distribution of amino acids in the corn steep liquor. Consequently, parameters for complex media constituents were estimated by averaging amino acid parameters over all 20 essential amino acids, which resulted in a carbon content of 3.69 and the N-molar degree of reduction of 18.59. Amino acids may not be completely equally distributed in the complex substrate; however, average nitrogen content and average degree of reduction are not expected to exhibit drastic changes for differing amino acid occurrences as many amino acids show similar values for the degree of reduction as well as the nitrogen content. Moreover, the validity of the assumption of equal distribution of amino acids was further affirmed by results from statistical validation as well as the closing of carbon, degree of reduction and nitrogen balances. For all substances, C-molar degree of reduction was calculated according to Eq. 15 and N-molar degree of reduction acc. to Eq. 16, respectively [17].

(15) $$xDOR_{C-molar}=\frac{Mol_{C}*4+Mol_{O}*(-2)+Mol_{H}+Mol_{S}*6}{Mol_{C}}$$

(16) $$xDOR_{N-molar}=\frac{Mol_{C}*4+Mol_{O}*(-2)+Mol_{H}+Mol_{S}*6}{Mol_{N}}$$

C-molar nitrogen content was calculated using Eq.17.

(17) $$xN_{C-molar}=\frac{Mol_{N}}{Mol_{C}}$$

N-molar carbon content of the complex substrate was calculated using Eq.18.

(18) $$xC_{N-molar}=\frac{Mol_{C}}{Mol_{N}}$$

Checking for gross errors was performed by comparing the actual error of calculated entities to the maximal expected error according to equations 19 to 21 resulting in the statistical test parameter *h*.

(19) E*x=ε

(20) $$h=\varepsilon^{T}*\Phi^{-1}*\varepsilon$$

(21) $$\Phi =E^{T}*\Psi *E$$

Equation 19 shows the calculation of residuals using actual and thus inherently noisy measurements, while for each individual rate the expected error is specified in the variance-covariance matrix *Ψ* of the rates which are assumed to be non-correlated. The statistical test parameter *h* is calculated according to equations 20 and 21 with *Φ* as the variance-covariance matrix of the residuals. Generally, the hypothesis of having accurately determined the system needs to be rejected if the *h*-value is higher than 2.71 (according to the chi-square distribution) with a degree of freedom of 1 (S = 1) and a confidence interval of 90% and 4.61 with a degree of freedom of 2 and 6.25 with a redundancy of 3, respectively. Thus, the above outlined approach allows judging raw data quality. Unless the test parameter *h* exceeds its threshold acceptance value, it can be assumed that the system was accurately determined. All data discussed in this study met the criteria assuming a maximum relative error of 5% during the exponential growth phase. This assumed maximum error was specified in the variance-covariance matrix *Ψ* for the rate vector.

For a detailed and exhaustive, step by step description of theoretical mathematical aspects of elemental balancing and methods for gross error checking, the reader is referred to benchmark studies within this field [18-20]. Finally, using the above outlined approach analytical demands were strongly reduced by inferring uptake kinetics of complex nitrogen components from mass balancing. Moreover, accurate and feasible strain and process characterization were facilitated.

### Batch cultivations

Preliminary test cultivations using the described system identified considerable blow-out of spores under submerse aeration conditions as the major factor inhibiting submerse growth on non-complex media and partly promoting adherent wall growth in the reactor headspace as well as on the top plate. As a result, top aeration was used for all cultivations until successful spore germination was confirmed by microscopic analysis, which was always in the range of 24–36 hours. Only then, the aeration regime was switched to submerse aeration. It is important to note, that unless blow-out of spores was successfully prevented, both fungal strains did not show any growth on defined media. Moreover, wall growth was effectively prevented for all cultivations by applying cooling tubes to the reactor headspace. We further complemented above outlined measures suggested in an earlier study [21] as we kept the liquid level constant by refilling broth volumes removed during sampling. Withdrawn broth volumes were complemented with defined media lacking carbon and nitrogen sources as repetitive sampling considerably lowered the liquid level and was identified to promote wall growth on the exposed reactor surface. Only the combination of submerse aeration and constant liquid level strategies enabled accurate determination of the biomass dry weight. As reactor and refill volume were recorded, water evaporation and broth dilution were corrected during data analysis, which was also reflected in the corrected concentrations of lactate remaining constant throughout the whole fermentation for cultivations containing complex media. Lactate in the fermentation media originated from corn steep liquor but was not consumed or produced by the fungi in the presence of excess carbohydrates. Consequently, for these cultivations, lactate could be used as an internal standard to double-check correction factors obtained from recorded balance weight values ( Additional file 1: Figure S1). The following subsections provide detailed discussion of screening cultivation results and an overview of key performance parameters is given in Table 1.

**Table 1:** Summary of Key Performance Parameters for all batch characterization studies

**Complex media fraction/Key performance parameter**	**Strain characterization on partly complex media**				**Verification on defined Media**	
	**BCB1 100%**	**P2 100%**	**BCB 125%**	**P2 25%**	**BCB1 0%**	**P2 0%**
**Duration [h]**	39.3	56.5	49.1	65.2	58	83.2
**Duration Lag-Phase [h]**	17	23	23	36	43	50
**μ**_**max**_**[h**^**-1**^**]**	0.18	0.13	0.15	0.16	**0.19**	0.16
**max. specific formation rate for gluconic acid [g·g**^**-1**^**·h**^**-1**^**]**	> 0.18	0	> 0.13	0	**0**	0
**max. specific formation rate for mannitol [g·g**^**-1**^**·h**^**-1**^**]**	> 0.03	0.03	0.05	0.03	0.06	0.06
**Formation of fructooligosaccharides**	GF_2_	GF_2-n_	**N.A.**	GF_2-n_	**N.A**.	N.A.
**Percentage of nitrogen content at the process end [%]**	100	100	**23**	33	N.A.	N.A.

(N.A. = not applicable).

#### Cultivation of BCB1 on 100% complex media

**Figure 2:**
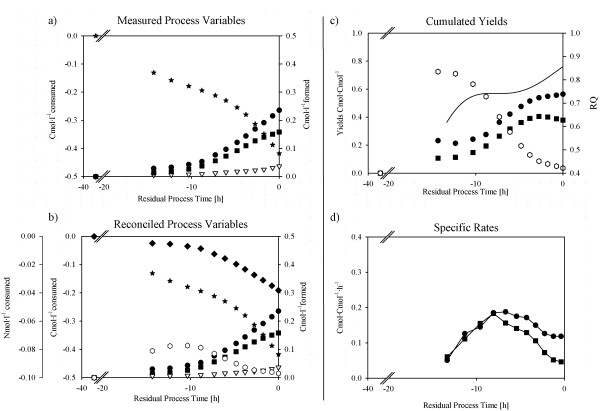
**Process performance charts for BCB1 batch cultivation on fully complex media.a**) **measured raw data.** (Dots), Biomass formed; (Stars), Initial substrate consumed; (Squares), Gluconic acid formed; (Open triangles down) Mannitol. **b**) **statistically verified measured raw data and calculated process variables.** (Dots), Biomass formed; (Stars), Initial substrate consumed; (Squares), Gluconic acid formed; (Open triangles down), Mannitol formed; (Open hexagons), GF_2_ formed; (Diamonds), Complex nitrogen consumed. **c**) **physiological dynamics including cumulated yields and RQ.** (Solid line), RQ; (Dots), Yield Biomass·Substrate^-1^; (Open hexagons), Yield GF_2_·Substrate^-1;^ (Squares), Yield Gluconic acid·Substrate^-1.^**d**) **specific formation rates for biomass and gluconic acid.** (Dots), specific growth rate; (Squares), q_p_ Gluconic acid.

When strain BCB1 was cultivated on 100% complex media, i.e. solely on complex N-sources, the total duration of the batch process was slightly below 40 h. An increase in the carbon dioxide evolution rate (CER) was first observed 17 h after inoculation indicating the transition from the lag-phase to the exponential growth phase (Table 1). Figure 2 illustrates measured process parameters and physiological dynamics such as specific rates and yields derived from mass balancing. Figure 2a shows raw data of measured components, whereas Figure 2b depicts statistically verified and reconciled process variables and calculated components, which allowed the deduction of physiological dynamics of biomass and side-product formation. By employing bespoken mass balancing approach, we were able to quantify the complex nitrogen components uptake kinetics as well as the formation of unwanted side-products. To identify the formed side-products and thus to fully understand the investigated system, we employed GC-MS analytics. We found that cultivation of strain BCB1 on fully complex media caused considerable formation of gluconic acid and mannitol as overflow-metabolites, as well as the formation of a trisaccharide containing a single glucose and 2 fructose moieties (hereafter called GF_2_). Formation of the chelating agent gluconic acid is a commonly known phenomenon causing acidification and thus benefits fungal growth in its natural habitat [22], and formation of mannitol might play a role as carbohydrate reserve and also protects the fungus against osmotic stress [23]. Interestingly, fructooligossacharide formation of higher order (i.e. GF_3_ – GF_X_), though reported for other *Penicillium chrysogenum* strains in literature [24], did not occur for strain BCB1 when cultivated on fully complex media. Only when all the unwanted side-products were taken into account, the mass balances based on carbon, degree of reduction and nitrogen (Eq. 2–21) closed. In fact, calculated and measured concentrations for GF_2_ matched over the exponential growth phase with an average relative standard deviation of approx. 8% ( Additional file 2: Figure S2). This underlines the possibility of quantifying oligosaccharide formation by mass balancing only, which makes time consuming analytical methods including identification by GC-MS, peak collection from chromatography and acid hydrolysis unnecessary.

As shown in Figure 2c, during the initial lag-phase, production of GF_2_ occurred up to an observed maximum yield of 0.7 Cmol·Cmol^-1^ initial substrate. The fructooligosaccharide was subsequently taken up during the exponential growth phase, whereas the gluconic acid yield increased until the end of the cultivation reaching a maximum of 0.4 Cmol·Cmol^-1^initial substrate. Mannitol on the other hand was produced to a minor extent with a significantly lower maximum yield compared to gluconic acid of approx. 0.08 Cmol·Cmol^-1^substrate, an observation which held true for all cultivations. Thus, strain characterization performed in this study focused on gluconic acid as the major side-metabolite. Generally, avoiding fructooligosaccharides and gluconic acid as unwanted side-products would be highly desired as their formation reduces the biomass space time yield and directly influences process performance. During the exponential growth phase the maximum specific growth rate was ~ 0.18 h^-1^ and the maximum formation rate of the major side-metabolite gluconic acid was approx. 0.18 Cmol·Cmol^-1^·h^-1^ (Figure 2d). The decrease in the specific formation rate for gluconic acid towards the end of the cultivation correlated with the increase of the respiratory quotient (RQ) and thus could be observed online in real-time (Figure 2c and 2d). The decrease in the specific growth rate from −10 h onwards (Figure 2d) might be explained by fungal biomass pellets having reached substrate diffusion limiting diameters and thus no longer exhibiting exponential growth characteristics [25].

Starting from cultivation on 100% complex media, the complex media fraction was reduced in the following experiments (*vide infra*). Consequently, strain characterization and process identification on fully complex media serves as benchmark analysis yielding the maximum rate vector *x* (Eq. 13), which is then used in mass balancing and data evaluation (Eq. 2–21).

#### Cultivation of BCB1 on 25% complex media

**Figure 3:**
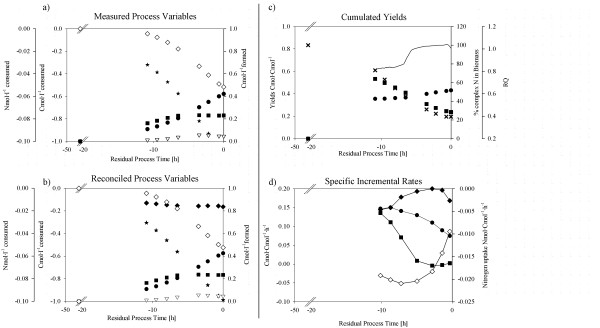
**Process performance charts for BCB1 batch cultivation on 25% complex media.a**) **measured raw data.** (Dots), Biomass formed; (Stars), Initial substrate consumed; (Squares), Gluconic acid formed; (Open triangles down), Mannitol formed; (Open Diamonds), NH_3_-N consumed. **b**) **statistically verified measured raw data and calculated process variables.** (Dots), Biomass formed; (Stars), Initial substrate consumed; (Squares), Gluconic acid formed; (Open triangles down), Mannitol formed; (Diamonds), Complex nitrogen consumed, (Open Diamonds), NH_3_-N consumed. **c**) **physiological dynamics including cumulated yields and RQ.** (Solid line), RQ; (Dots), Yield Biomass·Substrate^-1^; (Squares), Yield Gluconic acid·Substrate^-1^; (Crosses), Cumulated Percentage of Complex Nitrogen. **d**) **specific formation rates for biomass and gluconic acid.** (Dots), specific growth rate; (Squares), q_p_ Gluconic acid; (Diamonds), q_s_ complex nitrogen sources; (Open Diamonds), q_s_ NH_3_-N.

When cultivated on the 25% complex media formulation, strain BCB1 again formed gluconic acid and mannitol as side-products (Figure 3). However, production of gluconic acid occurred to a lesser extent compared to the cultivation on fully complex media with a maximum specific formation rate of 0.13 Cmol·Cmol^-1^·h^-1^ compared to 0.18 Cmol·Cmol^-1^·h^-1^ , respectively (Figure 2d and 3d). Formation of mannitol was again similar to the cultivation on fully complex media with a maximum yield of approx. 0.08 Cmol·Cmol^-1^ substrate. As the statistical data consistency check confirmed correct identification of the process without any gross errors, it was further verified that, in contrast to the fermentation on fully complex media, no fructooligosaccharides were formed (Figure 3a and 3b). Besides, redundant mass balancing allowed revealing dynamics of complex nitrogen components uptake characteristics. Specific nitrogen uptake rates exhibited a two-phase kinetic with the organism switching from growth on complex to growth on defined nitrogen sources approximately 5 hours prior to process end (Figure 3d). At the beginning of the cultivation, complex media constituents were excessively available and the fungus exhibited increased nutrient demands for germination and initial growth. However, once spores had successfully germinated, the culture switched to growth on defined nitrogen sources due to decreased nutrient demands. It was found that the percentage of nitrogen from complex sources of the total nitrogen incorporated in the biomass decreased over fermentation time (Figure 3c). At process end, 23% of the nitrogen incorporated in the biomass originated from complex media sources for BCB1 (POCN = 23%). This parameter was further used to characterize the strain specific potential to grow on fully defined media.

Kinetics of gluconic acid formation and complex nitrogen uptake showed highly similar trends suggesting a negative correlation between the production of the overflow metabolite and complex growth conditions (Figure 3d). Furthermore, the online detectable parameter of the RQ reflected kinetics of gluconic acid formation as it approached 1 as soon as production of gluconic acid was ceased (Figure 3c and 3d). As shown in Figure 3d, the specific uptake rate of complex nitrogen sources surprisingly increased again at the very end of the cultivation. We believe that due to the depletion of sucrose as available C-source (Figures 3a and 3b), the fungus resorted to the amino acids (i.e. complex N-sources) as alternative C-source.

Due to a prolonged duration of the lag-phase (23 hours compared to 17 hours for the cultivation on fully complex media; Table 1), the overall duration of the process was increased from 39.3 to 49 h, while the maximum specific growth rate was in a similar range (i.e. 0.18 h^-1^ and 0.15 h^-1^, respectively). As for the cultivation on fully complex media, the reduced specific growth rate at cultivation end compared to −10 h (Figure 3d) may be explained by fungal biomass pellets having reached substrate diffusion limiting diameters.

#### Cultivation of BCB1 on fully defined media

**Figure 4:**
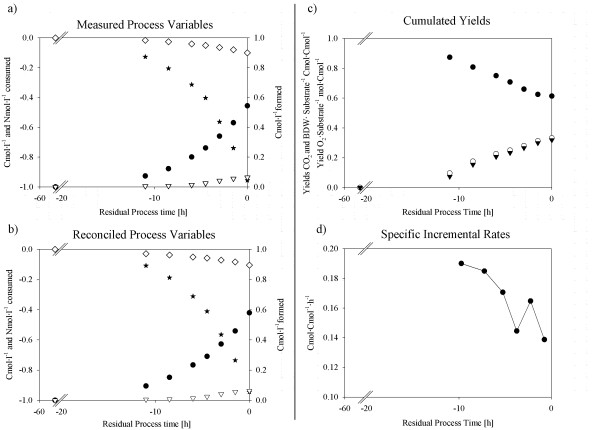
**Process performance charts for BCB1 batch cultivation on fully defined media.a**) **measured raw data.** (Dots), Biomass formed; (Stars), Initial substrate consumed; (Open triangles down), Mannitol formed; (Open Diamonds), NH_3_-N consumed. **b**) **statistically verified measured raw data and calculated process variables.** (Dots), Biomass formed; (Stars), Initial substrate consumed; (Open triangles down), Mannitol formed; (Open Diamonds), NH_3_-N consumed. **c**) **physiological dynamics including cumulated yields and RQ**. (Dots), Yield Biomass·Substrate^-1^; (Open dots), Yield O_2_·Substrate^-1^; (Triangles down), Yield CO_2_·Substrate^-1^. **d**) **specific formation rate for biomass.** (Dots), specific growth rate.

The cultivation on 25% complex media suggested several benefits for the strain BCB1 when switching from complex to defined media, namely preventing the formation of oligosaccharides and a reduced maximum specific formation rate of gluconic acid. These extrapolations from strain characterization on partly complex media were further verified in a cultivation on fully defined media. Lacking complex media constituents, production of gluconic acid was completely shut down and mannitol was identified as the only side-product (Figure 4a) formed at a maximum yield of approx. 0.08 Cmol·Cmol^-1^ substrate (Figure 4b). Again, validity of results was confirmed as *h*-values from statistical consistency checks were below the acceptable maximum. Despite the highest maximum specific growth rate of all cultivations exceeding 0.19 h^-1^ (Figure 4d), the overall duration of the process on fully defined media of 58 h was longer than for cultivations on 25% and 100% complex media due to an extended lag-phase of 43 h. Throughout the process, the RQ was 1 as yields for O_2_ and CO_2_ on substrate were nearly equal (Figure 4c). This correlated well with the physiological state of the culture, as activity was limited to biomass formation and only negligible formation of mannitol occurred, as discussed above (Figure 4a and 4b). Towards cultivation end, the specific growth rate decreased and the biomass yield concomitantly exhibited a decline while the yield for carbon dioxide increased (Figure 4c and 4d). These characteristics may be due to biomass pellets which no longer grew exponentially but according to the cube-root law [25,26].

#### Comparing strains BCB1 and P2

Within this study, both *P. chrysogenum* candidate strains BCB1 and P2 were characterized on 100% and 25% complex media in order to infer process performance on fully defined media. In addition, verification cultivations were performed on purely defined media for both fungal strains BCB1 and P2. Results from all cultivations are compared in Table 1.

##### Formation of unwanted side-products

Besides the desired production of biomass, also the formation of side-products such as gluconic acid, mannitol and oligosaccharides was observed for both strains, dependent on the used media formulation. However, strain P2 exhibited not only formation of the oligosaccharide GF_2_ but also higher oligosaccharides, *e.g.* GF_3-n_. While BCB1 ceased formation of oligosaccharides when reducing the complex media fraction from 100% to 25%, P2 still produced bespoken oligosaccharides (Table 1). Independent of cultivation conditions, P2 did not show any formation of gluconic acid. BCB1 on the other hand, produced gluconic acid in presence of complex media components. When switching to defined media however, production was stopped. Applying the proposed strategy, the following extrapolations from cultivations on partly complex media were verified in defined cultivations:

a) the specific production rate of gluconic acid can be minimized for strain BCB1 by reducing the fraction of complex media

b) formation of oligosaccharides can be prevented for strain BCB1 by reducing the fraction of complex media

Hence, starting from two candidate strains, the bioprocess could be successfully transferred to defined media conditions by selecting the more promising strain BCB1 using the outlined methodology. Finally, for the defined media cultivation of BCB1, the highest specific growth rate (0.19 g·g^-1^·h^-1^) was observed, while only very limited production of mannitol as the only side-product occurred (0.06 g·g^-1^·h^-1^).

##### Process Duration

For both strains, process duration increased with decreasing fractions of complex media. Considerably prolonged lag-phases were identified as the major causes for the increase in the overall batch duration (Table 1). In general, during germination the culture exhibits extended nutrient demands and thus benefits from complex media ingredients to a larger extent. However, we believe that the reduced demands for raw material quality control, as well as controllable, defined cultivation conditions outweigh the increase of the batch cultivation time on fully defined media. The actual process duration may not be influenced by the choice for defined media, because the process is at least a 2 stage process, for which equipment utilization and overall process productivity can be optimized by time and motion studies. Moreover, if, despite the possibility of timely staggering the usage of batch and fed-batch reactors and thus absorbing increased process duration for batch cultivations, the increased duration time of the batch process is a major issue, we suggest to implement a 3-stage process consisting of a complex media germination and a defined media batch and fed-batch stage.

##### Assimilation of the defined nitrogen source

The cumulated nitrogen fraction in the biomass at cultivation end originating from complex media constituents was considerably lower for BCB1 than for P2 when cultivated on 25% complex media (POCN of 23 and 33%, respectively). A consistent trend could be observed for the novel strain specific key parameter POCN (Eq.1) introduced in this study for both strains when cultivated on 25% complex and the verification runs on defined media. Therefore, the suggested key parameter allowed quantification of the strain specific affinity towards defined nitrogen sources and thus an easy identification of the most promising strain for switching the process from complex to defined cultivation conditions. The increased affinity of strain BCB1 to defined nitrogen sources on partly complex media, together with the reduced formation of oligosaccharides (Table 1 and Figure 3c and 4c) identified BCB1 to be superior over P2 for switching the process to defined cultivation conditions. For this reason, a fully defined 2-stage fed-batch production process was performed with strain BCB1.

### Fully defined 2-stage production process for strain BCB1

**Figure 5:**
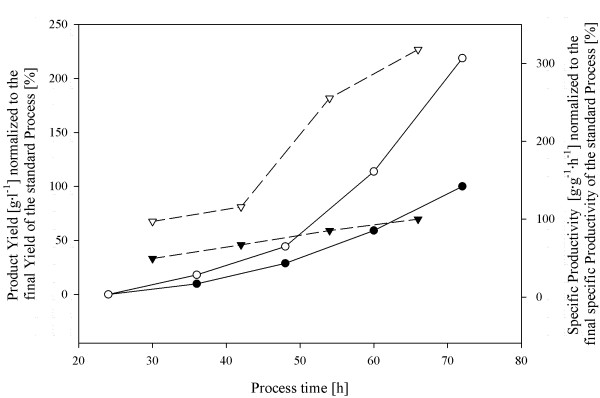
**Key performance parameters for a 2-stage industrial penicillin production process at defined and complex batch cultivation conditions.** (Dots), Normalized product yield using complex media; (Open dots), Normalized product yield using defined media; (Triangles down), Normalized specific productivity using standard complex media; (Open Triangles down), Normalized specific productivity using defined media.

Having identified BCB1 as the more promising candidate strain, we performed additional fed-batch verification runs with batch cultivations on either complex or defined media in order to compare specific penicillin V productivity and product yields for both cultivation strategies. The preceding batch cultivations further confirmed results from previously conducted, initial screening cultivations demonstrating the validity of screening results. In Figure 5, the comparison of fed-batch key performance parameters for the respective cultivation regimes is shown. Very interestingly, results showed that conventional, complex media production processes could be significantly outperformed by switching a previous 2 stage complex/defined production process to a 2 stage fully defined strategy since the final product yield was more than doubled. Moreover, a 3-fold increase of the specific production rate for the defined cultivation conditions suggested an even higher increase for the product yield at prolonged process duration. Underlying reasons for improved process performance at fully defined process regime may include that the physiological activity in the initial batch stage was limited to biomass growth only, and formation of unwanted side-products could be avoided. Additionally, the fact that the culture did not need to adjust to altered cultivation conditions when transferred to the second stage may had a beneficial effect. Hence, we could show, that for production processes comprising of several stages employing both, complex and defined media compositions, the here presented approach may not only be used to switch the process to fully defined media but also to significantly increase product yields and to successfully circumvent problems resulting from variations in complex raw materials.

## Conclusions

The applied strategy for strain characterization of filamentous fungi on partly complex media at different complex/defined media ratios using redundant mass balancing techniques facilitated the elucidation of physiological kinetics for complex nitrogen species uptake mechanisms and growth characteristics on complex media. Interdependencies of specific biomass and side-metabolite production rates, formation of fructooligosaccharides, specific complex media components uptake rates and fungal strains were revealed. The novel strain specific key parameter “percentage of complex nitrogen of the overall nitrogen uptake (POCN)” enabled straight-forward identification of the most promising strain for switching the process from complex to defined conditions. Strain characterization on combined complex/defined media only asked for limited analytical methods and thus allowed maximizing the overall industrial objectives of increasing both, method throughput and the generation of scientific process understanding.

If a bioprocess engineer can chose from a collection of fungal candidate strains for switching production processes from complex to defined media, we therefore suggest the following procedure:

 Strain characterization and process identification on fully complex media for benchmark analysis of the system’s maximum complexity. Cultivations should be conducted with top aeration during germination as well as cooling tubes at the reactor headspace and refilling sampling volumes to reliably determine the fungal biomass dry weight. Subsequent and easy-to-do mass balancing based on the maximum rate vector *x* identified in this step then allows the elucidation of complex nitrogen components uptake as well as side-product formation kinetics.

Batch cultivations on 25% complex media to determine strain specific uptake rates for complex and defined nitrogen compounds and the percentage of complex nitrogen of the overall nitrogen uptake (POCN). The lower the identified POCN, the more suitable is the strain for switching the process from complex to defined media.

Switching the process to a defined cultivation strategy with the most promising fungal strain.

Applying this approach, we obtained a 2-fold increase of the overall penicillin space time yield and a 3-fold increase in the maximum specific penicillin formation rate with the candidate strain BCB1 on fully defined media formulation compared to a 2 stage complex/defined fermentation strategy. Consequently, this approach is not only particularly suitable for industrial strain characterization applications where throughput and time are of major concern, but can also lead to more efficient bioprocesses.

## Methods

### Strains

Spore suspensions of two *P. chrysogenum* candidate strains for penicillin production (code BCB1 and P2), which are proprietary, engineered strains, were kindly provided by Sandoz GmbH (Kundl, Austria).

### Cultivation system

The investigated bioprocess displayed an industrial scale penicillin production process and comprised a batch fermentation on complex media with corn steep liquor as nitrogen source followed by a fed-batch process for penicillin production using a defined media feeding strategy and ammonium sulfate as defined nitrogen source.

#### Batch cultivations

The complex media was based on the media used in [27] and consisted of: Sucrose 18 g·l^-1^, Glucose 3 g·l^-1^, Corn steep liquor 26 g·l^-1^, Silicone Oil 1 ml·l^-1^. Composition for the defined media was as follows: Glucose 30 g·l^-1^, (NH_4_)_2_SO_4_ 8.75 g·l^-1^, KH_2_PO_4_ 1.6 g·l^-1^, NaNO_3_ 0.2 g·l^-1^, KCl 0.5 g·l^-1^, CaCl_2_ × 2H_2_O 0.067 g·l^-1^, MgSO_4_ × 7H_2_O 0.5 g·l^-1^, TES-Stock 10 ml·l^-1^, Silicone Oil ml·l^-1^. TES-Stock consisted of: EDTA 14 g·l^-1^, CuSO_4_ × 5H_2_O 0.5 g·l^-1^, ZnSO_4_ × 7H_2_O 2 g·l^-1^, MnSO_4_ x H_2_O 2 g·l^-1^, FeSO_4_ × 7H_2_O 4 g·l^-1^. For both strains, characterization was performed in combined complex/defined batch cultivations at 100%, 25% and 0% complex media content. With a determined amino acid content of approx. 25% [w·w^-1^ for the corn steep liquor used in this work the C-molarity of batch media was always in the range of 0.85 to 1 Cmol·l^-1^.

Batch fermentations were performed in a 1.8-l stirred bioreactor (Applikon, The Netherlands), with the actual working volume of 1.5 liters. Cultures were aerated through a standard single port sparger located below the stirrer at a constant aeration rate of 0.8 vvm. Aeration air was sterilized through PTFE air filters of 0.2 μm pore size (Whatman, UK). Aeration flow was kept constant using the mass-flow-controller system 2Proc (Aalborg, USA). To prevent blow-out of spores, mode of aeration was switched from headspace to submerse after completed germination only. Water stripping from the reactor was prevented by the use of an off-gas condenser kept at a temperature of 8°C by means of a cryostat. The temperature of the culture was kept at 25°C by an external heat jacket, which was heated/cooled using a thermo circulator. The pH of the culture was strictly kept at 6.5 +/− 0.1 by addition of 2.5 N NaOH. Dissolved oxygen tension and pH were measured using a sterilizable pO_2_ and pH probe, respectively (both Mettler-Toledo, USA). Agitation was performed using three six-bladed Rushton turbine impellers. Rotation speed was controlled in order to guarantee sufficient dissolved oxygen tension. Throughout all experiments, the dissolved oxygen tension was controlled at 40% by adjusting the agitator speed. All process control measures were performed by the integrated process control and management system Lucullus (Biospectra AG, Switzerland). Fermentations were inoculated with spores from rice cultures. For all cultivations inoculum concentration of living spores was 1.2 × 10^9^ l^-1^. Foaming was prevented by addition of small quantities of PPG 2000 (up to 1 ml).

The investigated complex media can be considered a typical empirically optimized, industrially used batch media for *Penicillium spp.* Addition of corn steep liquor accelerates germination of the fungus and thus reduces the lag-phase. Glucose was replaced with sucrose as the major carbon source in order to suppress significant formation of gluconic acid [28], however presence of sucrose is known to cause oligosaccharide formation [29,30].

#### Fed-batch cultivations

Fed-batch cultivations were carried out in a 7.5-l stirred bioreactor in defined growth media (Infors, Switzerland). Media composition was as follows and based on the media used in [31]: Glucose 1 g·l^-1^, (NH_4_)_2_SO_4_ 7 g·l^-1^, KH_2_PO_4_ 1.6 g·l^-1^, FeCl_3_.6H_2_O 0.02 g·l^-1^, MgSO_4_.7H_2_O 0.1 g·l^-1^, KCl 0.5 g·l^-1^, PPG 2000 0.05 ml·l^-1^, CuSO_4_.5H_2_O 5.5 mg·l^-1^, ZnSO_4_.7H_2_O 35.6 mg·l^-1^, MnSO_4_.H_2_O 29.5 mg·l^-1^, CaCl_2_.2H_2_O 65 mg·l^-1^. Glucose was fed at 2.65 ml·l^-1^·h^-1^with a feed concentration of 500 g·l^-1^. Feeding of a 200 g·l^-1^ (NH_4_)_2_SO_4_ solution, representing the N-source in defined media, was started after 5 h with a rate of 0.9 ml·l^-1^·h^-1^, was subsequently set to 1.5 ml·l^-1^·h^-1^ from 15–50 h, to 1.35 ml·l^-1^·h^-1^ from 50–70 h and to 1.2 ml·l^-1^·h^-1^from 70 h until process end. Sodiumphenoxyacetate as Penicillin V precursor was fed at 0.4 ml·l^-1^·h^-1^ with a feed concentration of 160 g·l^-1^. Process duration was 72 hours. Fed-batch processes were inoculated with 160 ml/l cultivation broth from batch processes as soon as carbohydrate exhaustion was indicated by an pH increase of 0.5. Cultures were aerated through a standard multiport sparger located below the stirrer at a constant aeration rate of 1.35 vvm (with respect to the starting volume). The pH of the culture was kept at 6.5 +/− 0.1 by addition of 5 N NaOH and 15% H_2_SO_4_; otherwise fermentation set-up and control was identical to batch cultivations (*vide supra*).

### Analytical methods

Determination of biomass dry weight was performed in duplicates by pressure aided filtering of 10 ml of culture broth on pre-dried and pre-weighted Pall glass fiber filters type A/E (Pall, USA). Prior to drying, filters were washed twice with 20 ml deionized H_2_O for cultivations on defined media, or, when beneficial for removal of insoluble media constituents, with 5 ml 2% HCl and 5 ml Acetone first, secondly with 5 ml Acetone and thirdly with 10 ml H_2_O.

Analysis of glucose, TCA-intermediates, organic acids and sugar alcohols was performed by high pressure liquid chromatography, using an Agilent Technologies Series 1100 HPLC with RI and DAD detector (Agilent, USA) via isocratic elution with 0.1% H_3_PO_4_ on a SUPELCOGEL™ C-610 H column (Supelco, Sigma-Aldrich, USA).

Detailed quantitative analysis and identification of organic components present in the fermentation broth (including organic acids, carbohydrates and di/tri-saccharides) was performed by an Agilent Technologies 7890a gas-chromatography system coupled to a 5975 C XL MSD mass-spectrometer (Agilent, USA). Samples were dried in the vacuum oven for 20 min at 40°C. 200 μl pyridine were added for quantitative dissolution. Samples were silylated with 150 μl hexamethyldisilazan (HMDS) and 70 μl trimetyl-chlorsilan (TMCS). Samples were then shaken for dissolution while bigger aggregates were split in an ultra-sonic bath. Derivatization was performed at least for 4 hours, preferably overnight. 100–150 μl were subsequently transferred to GC vials using insert tubes. Analysis was performed on a Helium Column: HP-5, length: 30 m, diameter: 0.32 mm, 0.25 μm film thickness using the following temperature program: 150°C for 1 min; increase to 220°C at 4°C·min^-1^, then to 320°C at 20°C·min^-1^. Hold at 320°C for 6.5 min. Injector 260°C, Detector 300°C. Split 10:1. Injection volume 1 μl. Average velocity: 30 cm·sec^-1^. Oligosaccharides were also analyzed as their monomeric constituents using the above described method after acid hydrolysis in 4 M H_2_SO_4_ at 100°C for 4 hours.

Analysis of penicillin V and phenoxyacetic acid was performed by high pressure liquid chromatography using a ZORBAX C-18 Agilent Column (Agilent Technologies, USA) and 28% ACN, 6 mM H_3_PO_4_, 5 mM KH_2_PO_4_ as elution buffer.

Quantitative enzymatic analysis of ammonia was performed using a commercially available assay (Randox, USA) on the Cubian XC enzymatic robot (Innovatis, Roche Diagnostics, Switzerland).

Biomass elemental composition needed for stoichiometric balancing was analyzed at two time points for all cultivations by determining C, H, N and O content at the Microanalytical Laboratory of the University of Vienna (Austria).

For all offline analyses, samples were immediately put on ice after withdrawal from the reactor and stored at - 20°C until further analysis for a maximum duration of two weeks.

## Abbreviations

CER: Carbon dioxide evolution rate; CPP: Critical process parameters; CQA: Critical quality attributes; DOR: Degree of reduction; E: Elemental matrix; ε: Residue vector; h: *h*-value, statistical test value; ICH: International conference on harmonization; K: Number of balances; KPP: Key performance parameters; M: Number of measured rates; N: Overall number of rates; *N*_*n*,*IN*/*OUT*_: Amount of species n consumed (*IN*) or produced (*OUT*); POCN: Percentage of complex nitrogen; QbD: Quality by Design; *R*_*n*_: Recovery coefficient for species *n*; RQ: Respiratory quotient; S: Degree of redundancy; *X*: Biomass; *x*: Measurement vector; xDOR: c-molar degree of reduction for biomass; xN: c-molare nitrogen content for biomass; *Φ*: variance-covariance matrix of the residues; *Ψ*: variance-covariance matrix of the rates.

## Competing interests

The authors declare that they have no competing interests.

## Author’s contribution

AEP and CH designed the study. AEP performed the study including cultivations, fermentation analysis and data evaluation. AEP and OP wrote the manuscript. CH supervised the study. All authors read and approved the final manuscript.

## Supplementary Material

Additional file 1 **Figure S1.** Corrected concentrations for the corn steep liquor component lactate and biomass dry weight using the example of strain BCB1 cultivated on 25% complex media. (Open dots), dilution corrected concentration of lactate; (Closed dots), dilution corrected concentration of biomass dry weight. Lactate is not consumed and neither produced by the fungus in the presence of carbohydrates. The corrected concentration remained constant throughout the fermentation, underlining the successful correction for dilution effects. Click here for file

Additional file 2 **Figure S2.** Calculated concentrations of GF_2_ vs. measured concentrations. (Dots), measured concentration of GF_2;_ (Open Dots), Calculated concentration of GF_2_ from mass balancing. Click here for file
